# International expert consensus on the management of allergic rhinitis (AR) aggravated by air pollutants: Impact of air pollution on patients with AR: Current knowledge and future strategies

**DOI:** 10.1016/j.waojou.2020.100106

**Published:** 2020-04-03

**Authors:** Robert Naclerio, Ignacio J. Ansotegui, Jean Bousquet, G. Walter Canonica, Gennaro D'Amato, Nelson Rosario, Ruby Pawankar, David Peden, Karl-Christian Bergmann, Leonard Bielory, Luis Caraballo, Lorenzo Cecchi, S. Alfonso M. Cepeda, Herberto José Chong Neto, Carmen Galán, Sandra N. Gonzalez Diaz, Samar Idriss, Todor Popov, German D. Ramon, Erminia Ridolo, Menachem Rottem, Wisuwat Songnuan, Philip Rouadi

**Affiliations:** a Johns Hopkins School of Medicine, Baltimore, MD, USA; b Department of Allergy & Immunology, Hospital Quironsalud Bizkaia- Bilbao, Spain; c INSERM U 1168, VIMA: Ageing and Chronic Diseases Epidemiological and Public Health Approaches, Villejuif, France; d University Versailles St-Quentin-en-Yvelines, France; e Humanitas University & Research Hospital, Milano, Italy; f Division of Respiratory and Allergic Diseases, High Specialty Hospital A. Cardarelli, Napoli, Italy; School of Specialization in Respiratory Diseases University Federico II Naples, Italy; g Pediatric Respiratory Medicine Division, Complexo Hospital de Clinicas, UFPR, Curitiba, Brazil; h Dept. of Pediatrics, Nippon Medical School, Tokyo, Japan; i UNC Center for Environmental Medicine, Asthma, and Lung Biology; Division of Allergy, Immunology and Rheumatology, Dpt. of Pediatrics UNS School of Medicine, USA; j Allergy-Centre-Charité, Charité–Universita¨tsmedizin Berlin, Berlin, Germany; k Medicine & Ophthalmology Hackensack Meridian School of Medicine at Seton Hall University Nutley, New Jersey, USA; l Institute for Immunological Research, University of Cartagena, Cartagena de Indias, Colombia; m Centre de Bioclimatology, University de Florence, Florence, Italy; n SOS Allergy and Immunology, Prato - USL Toscana Centro, Italy; o Fundación Hospital Universitario Metropolitano de Barranquilla, Barranquilla, Colombia; p Federal University of Paraná, Brazil; q Department of Botany, Ecology and Plant Physiology, University of Córdoba, Spain; r San Francisco Centro de Especialistas Médicos Monterrey NL, Mexico; s Department of Otolaryngology- Head and Neck Surgery, Eye and Ear University Hospital, Beirut, Lebanon; t Alexander's University Hospital Clinic of Allergy & Asthma, Bulgaria; u Alergia e Inmunología, Hospital Italiano Regional del Sur, Bahía Blanca-Buenos Aires, Argentina; v Department of Clinical and Experimental Medicine, Università; di Parma, Parma, Italy; w Allergy Asthma and Immunology, Emek Medical Center, Afula, Israel; x Rappaport Faculty of Medicine Technion, Israel Institute of Technology, Haifa, Israel; y Department of Plant Science, Faculty of Science, Mahidol University, Bangkok, Thailand; z Systems Biology of Diseases Research Unit, Faculty of Science, Mahidol University, Bangkok, Thailand

**Keywords:** Allergic rhinitis, Occupational rhinitis, Air pollution, Climate change, Air pollutants, Indoor air quality, Oxidative stress, Antioxidant enzymes, AP, Activator protein, AER, Allergic eosinophilic rhinitis, AR, Allergic rhinitis, ARE, Antioxidant response element, CO, Carbon monoxide, CFS, Chronic fatigue syndrome, COPD, Chronic obstructive pulmonary disease, DAMP, Damage-associated molecular patterns, DEP, Diesel exhaust particles, ECAT, Elemental carbon attributable to traffic, ECP, Eosinophil cationic protein, GSH-Px, Glutathione peroxidase, HVAC, Heating, ventilation and air conditioning, HO, Hemeoxygenase, HEPA, High efficiency particulate air, HDM, House dust mites, IAP, Indoor air pollution, IAQ, Indoor air quality, INS, Intranasal steroids, LDH, Lactate dehydrogenase, MSQPCR, Mold specific quantitative PCR, MCP, Monocyte chemotactic protein, NOx, Nitric oxides, NO_2_, Nitrogen dioxide, NAR, Non allergic rhinitis, Nrf_2_, Nuclear factor erythroid-2 related factor, NF-κβ, Nuclear factor kappa β, OAP, Outdoor air pollution, O_3_, Ozone, PON, Paraoxonase, PM, Particulate matter, PAMP, Pathogen-associated molecular patterns, RNS, Reactive nitrosative species, ROS, Reactive oxygen species, SO_2_, Sulphur dioxide, SOD, Superoxide dismutase, TLR, Toll like receptor, TOS, Total oxidative status, TRAP, Traffic related air pollutants, TNF, Tumor necrosis factor, UFP, Ultra-fine particles, VOCs, Volatile organic compound

## Abstract

Allergic rhinitis affects the quality of life of millions of people worldwide. Air pollution not only causes morbidity, but nearly 3 million people per year die from unhealthy indoor air exposure. Furthermore, allergic rhinitis and air pollution interact. This report summarizes the discussion of an International Expert Consensus on the management of allergic rhinitis aggravated by air pollution. The report begins with a review of indoor and outdoor air pollutants followed by epidemiologic evidence showing the impact of air pollution and climate change on the upper airway and allergic rhinitis. Mechanisms, particularly oxidative stress, potentially explaining the interactions between air pollution and allergic rhinitis are discussed. Treatment for the management of allergic rhinitis aggravated by air pollution primarily involves treating allergic rhinitis by guidelines and reducing exposure to pollutants. Fexofenadine a non-sedating oral antihistamine improves AR symptoms aggravated by air pollution. However, more efficacy studies on other pharmacological therapy of coexisting AR and air pollution are currently lacking.

## Introduction

Pollution is the introduction of excessive elements into the environment resulting in detrimental health effects. It can take the form of chemical substances or energy, such as noise, heat, or light. It can contaminate air, soil and water, and it can originate from naturally occurring contaminants or from human engineered activities. It is estimated that in 2015 pollution accounted for the demise of 9 million people worldwide of which household air pollution was responsible for 2.9 million deaths.^1^

**Fig. 1:**
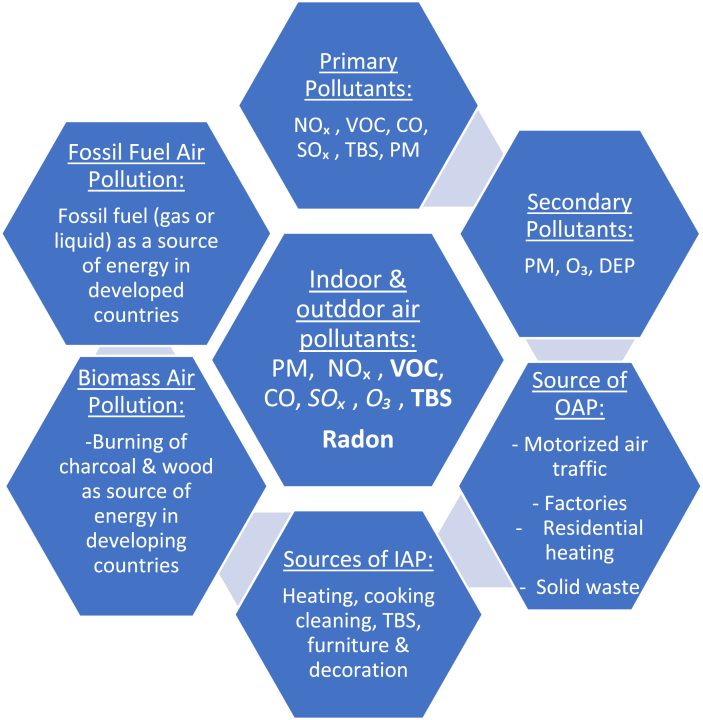
Sources and components of indoor/outdoor air pollution. Bold and *Italic* words denote pollutants more important in IAP and OAP, respectively. Adapted from https://www.who.int/airpollution/ambient/pollutants/en/. Published 2017^2^

Air pollution (AP) relates to substances emitted above permissible levels in ambient air. Chemical air pollution is generated by solid (particulate), liquid, or gaseous emissions. Based on their source and derivation, these pollutants can be classified into indoor or outdoor, primary (if directly emitted into the atmosphere), or secondary (if these react or interact therein, e.g. ozone-O_3_) pollutants. Biological air pollution is partly caused by aeroallergens which can preferentially contribute to indoor or outdoor atopic illnesses such as allergic rhinitis (AR) and asthma^2^ (see Fig. 1).

Allergic rhinitis (AR) and asthma are IgE mediated type 1 hypersensitivity illnesses triggered by a spectrum of environmental allergens like pollen (mainly outdoor origin), arthropod- or mammalian-derived allergens (mainly indoor origin) such as dust mites, cockroaches, cat allergens or molds.^3^ Other rhinitis phenotypes can have an allergic, non-allergic, or mixed inflammatory profile, such as that triggered by irritants or occupational allergens in a particular work environment (occupational rhinitis); or can be neurogenic non-inflammatory rhinitis (vasomotor rhinitis).^4^^,^^5^

Studies on the association between exposure to (indoor/outdoor) air pollution and prevalence of atopy in children and adults have yielded mixed results, partly due to differences in epidemiological study design, methods of exposure assessment, and duration of exposure.^6^, ^7^, ^8^, ^9^ Furthermore, although numerous studies have linked exacerbation of AR and asthma with rising levels of air pollutants,^10^, ^11^, ^12^, ^13^, ^14^ some did not.^15^^,^^16^ Moreover, there is a growing body of literature on the mechanistic effect of pollutants on atopic and healthy individuals and animal models favoring an oxidative stress-mediated non-allergic inflammatory pathway (see below); however, not all studies are supportive of such pollutant-induced non-allergic inflammation.^17^, ^18^, ^19^, ^20^ This could be partly related to the variable and complex composition of pollutants and the heterogeneous nature of clinical models.

The previous decades witnessed an epidemic of allergic diseases whose manifestations and severity are aggravated by climate factors and air pollution.^21^ An outstanding challenge is to understand the development of AR and pollution. The challenge lies in recognizing the individual external environmental exposures (external exposomes) and their effect at the cellular levels (internal exposomes). Thus, *specific* external exposomes, exemplified by air pollution, aeroallergens, as well as *non-specific* external exposomes, such as climate change, interact with internal exposomes to produce different phenotypic expression of organ-specific atopic diseases. Understanding how these environmental factors influence allergy development and the status of existing disease is crucial to stipulate counteractive management modalities.^22^

## Timing of pollutant exposure and impact on health

Epidemiological studies suggest timing of exposure to pollution as early as pre-conceptional period can impact allergic diseases later in life. In support of this concept, a questionnaire-based study involving a large number of preschool children reported an increased risk of childhood atopic diseases, mainly asthma, allergic rhinitis, and eczema when exposed to ambient industrial and traffic-related air pollutants (TRAP) during perinatal period.^23^ A prospective study involving children exposed to TRAP at birth and followed till the age of 4, diesel exhaust particles (DEP) exposure at age 1 was positively associated with aeroallergen sensitization at ages 2 and 3.^24^ Also, pregnant women, when exposed to biomass smoke (wood burning in developing countries) during their gestational period are at a higher risk of low birth weight and still birth with an attributable population risk of 21% and 26.3%, respectively.^25^ Other outdoor pollutants exposure such as NO_2_ increases risk of early childhood respiratory tract disease.^26^

## Outdoor air pollution (OAP)

Outdoor air pollution (OAP) constitutes more than 3% of the annual Disability-Adjusted Life Year (DALY) in 2010.^27^ Important outdoor pollutants such as airborne particulate matter (PM), O_3_, TRAP, and DEP, among others pose significant health risks.

PM in its solid (black carbon and metals) or liquid (nitrates, sulphates, ammonium salts) form,^28^, ^29^, ^30^, ^31^ can be anthropogenic, or produced naturally during dust storms, forest fires or infrequently during volcanic eruption.^32^ PM can be of changing composition,^33^ and have variable aerosol size-airway distribution.^27^ For example, extra-thoracic (nose and throat) deposition relates to PM ranging in size from 2.5 μm to 10 μm, while deposition of inhaled PM less than 2.5 μm occurs in the lung.^34^ Along this, PM can be also classified into ultrafine (<0.1 μm), fine (0.1–2.5 μm), or coarse (2.5–10 μm) particles depending on their size. Ultra-Fine Particles (UFP), sub-micron scale particles generated through the use of nanotechnology, have larger surface area than that of larger sized PM.^35^ Thus, they can act as carrier vehicles for metals and other organic compounds. In addition to this, both *in vivo* and *in vitro* studies suggest ultrafine and fine particles are more important in causing lower airway pathology.^36^ Still various individual factors modulate airway distribution and deposition of PM such as maturation and integrity of respiratory tract, general health, and, importantly, nasal versus oral breathing.^37^

Irrespective of allergen sensitization, experimental PM exposure can trigger oxidative pathways (see section Mechanism of Oxidative stress in healthy humans and animals), exacerbates allergic airway disease and increase organ responsiveness (see section Mechanism of Oxidative stress in AR patients).

*Ground level O*_*3*_ is recognized as one of the most injurious air pollutants to mankind and ecosystems,^38^^,^^39^ and it is produced from precursors like nitrogen oxides (automobile exhaust) and volatile organic compounds (VOCs) in the presence of sunlight. Ecological associations of ambient O_3_ and AR, asthma, and atopic dermatitis have been demonstrated.^40^^,^^41^ In experimental humans and animal models, exposure to ozone impairs pulmonary function, increases airway responsiveness, and induces lower airway inflammation.^42^ Although some epidemiological data have suggested a role for short-term exposure to ozone in triggering asthma, as determined by hospitalizations and emergency room visits,^43^ evidence regarding the impact of long-term exposure to ozone in asthma has been inconsistent.^44^ At the cellular level, ozone can trigger epithelial cellular membrane to discharge cytokines and arachidonic acid metabolites such as cyclooxygenase and lipoxygenase derivatives. In addition to this, ozone can decrease indirectly mucociliary clearance and free radicals production.^42^

*TRAP* is ubiquitous and complex in structure composed of traffic markers, in their solid or gaseous phases, namely black carbon from diesel exhaust, gases like nitrous oxides and carbon monoxide, originating from general traffic and petrol exhaust, respectively. Other constituents are metals like zinc and copper originating from breaks and tires, respectively.^45^ Nitrogen dioxide contributes to ground level ozone formation and has an inflammatory effect on the respiratory tract.^46^ Along with this, in one longitudinal retrospective study conducted between 1997 and 2006, the decrease in nitrogen dioxide and other air quality parameters such as PM and carbon monoxide correlated with decrease in yearly prevalence of AR.^47^ Moreover, epidemiologic studies suggested TRAP depends on traffic factors confined to an area within 200 m^2^ of home residence, namely proximity from concentration of main roads.^48^ Also, petroleum distillates able to release SO_2_ are associated with acute respiratory symptoms in children living in close proximity to the industrial sources.^49^

*DEP* has a solid aggregate of elemental carbon and metals, in addition to a gaseous phase composed in its majority of non-toxic inorganic gases such as oxygen and nitrogen. Organic components of DEP such as benzene, pyrenes, and others, are collectively termed poly-aromatic hydrocarbons, or PAHs.^50^

Based on epidemiological data, WHO and the International Agency for Research on Cancer have classified DEP as highly carcinogenic to humans.^51^^,^^52^ The suggested pulmonary genetic damaging effects and inflammatory toxicity, whether mediated by oxidative stress or not, have been described elsewhere,^50^ keeping in mind that simulation of real-life exposure conditions in experimental studies seems difficult to achieve due to high complexity of DEP. Furthermore, climate chamber studies involving both ragweed and house dust mite (HDM) allergic patients suggested a synergistic effect of DEP on atopic inflammatory markers following respective allergen challenge and exposure.^53^ Taken all together these data suggest a significant impact of DEP on respiratory organs in atopic and non-atopic subjects. Contrary to this, a well-designed birth cohort study examining the risk factors predictive of AR at age 3 failed to demonstrate an association between DEP exposure, as represented by elemental carbon attributable to traffic (ECAT), and atopic status.^54^ Also, earlier epidemiological data from Japan revealed traffic related increase in prevalence of Japanese cedar pollinosis irrespective of cedar pollen level.^55^ These conflicting results could be attributable to differences in study designs and protocols, different exposure assessment or the discrepancy in impact of each studied DEP "representatives" on atopic status when examined separately, thus underlying the complex physical nature of DEP.

This overall trafficking and subsequent settling of pollutants in ambient air is affected by climate factors (temperature, humidity), weatherization (building energy-efficient airtight houses), indoor ventilation/activities, and other determinants of ambient pollutant concentration.

## Indoor air pollution (IAP) and indoor air quality (IAQ)

**Fig. 2:**
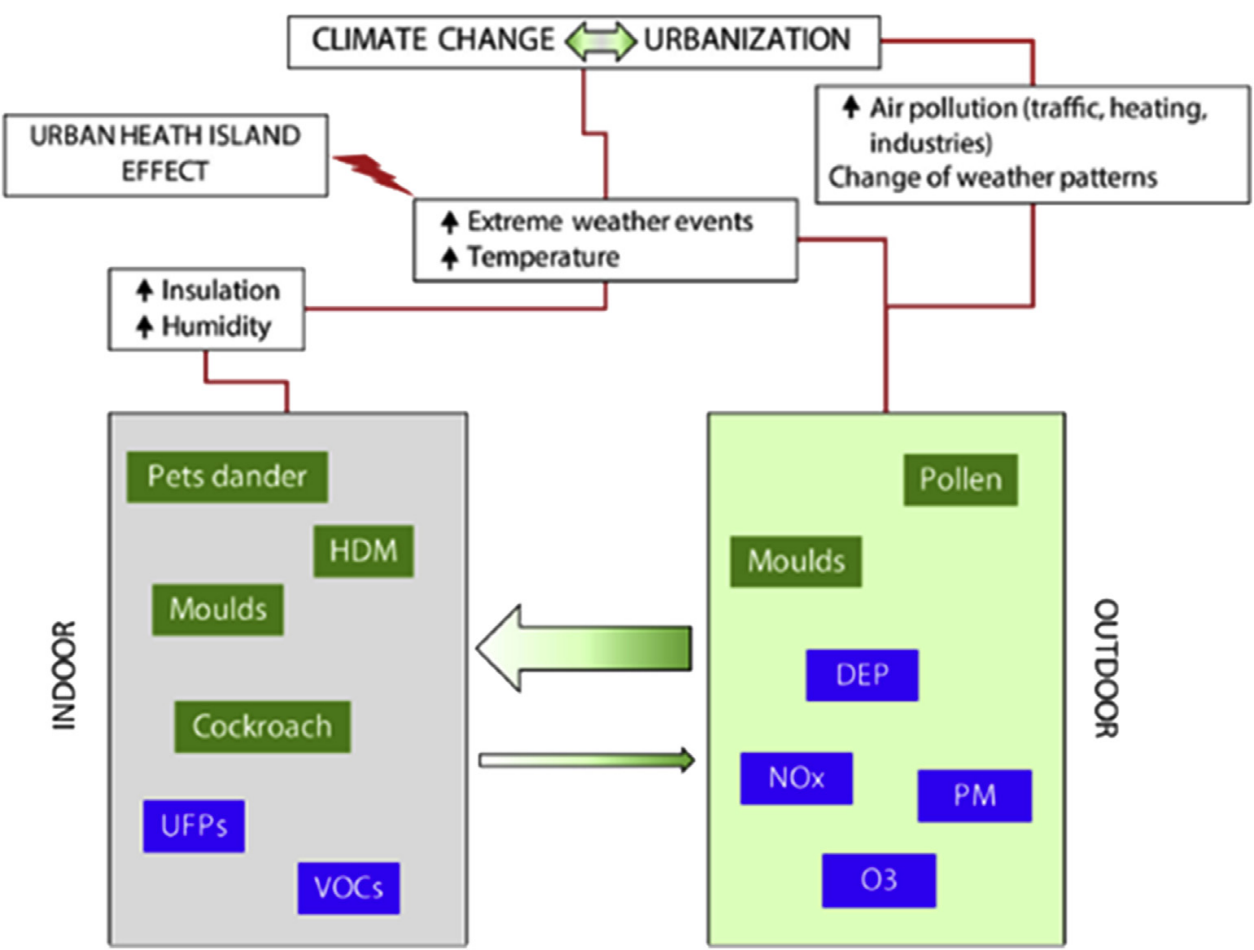
Indoor and outdoor exposures to aeroallergens and air pollutants and environmental factors affecting their production and concentrations. Diesel exhaust particles (DEP), Nitrogen oxides (NOx), Particulate matter (PM), Ground level ozone (O_3_), House dust mite (HDM), Ultrafine particles (UFPs), Volatile organic compounds (VOCs). From Cecchi L et al. J Allergy Clin Immunol. 2018; 141(3):846–857. https://doi.org/10.1016/j.jaci.2018.01.016^22^

Focusing on indoor environments where we mainly live, IAQ is important to understand since it defines total human exposure to chemical air pollutants, keeping in mind OAP and IAP overlap in some pollutants, most notably particulate matter (PM) and gaseous pollutants (volatile organic compounds - VOCs).^56^ IAQ is altered by infiltrating outdoor air, air ventilation, and indoor pollutants, in addition to interactions between building system/construction techniques and occupants (see Fig. 2).^22^ For example, the new urban building construction designs aimed at indoor energy conservation by building air-tight residences (weatherization) resulted in reduced outdoor air streaming into the home or reduced air exchange between indoor and outdoor environments with subsequent increased levels of indoor contaminants (2-5 fold), when compared to outdoor ambient levels.^57^ Conversely, IAQ may be compromised by pollutants usually present at low level indoors yet have a significant health damaging effect due to prolonged exposure. Taken together, IAQ and its impact on health relies on estimation of specific pollutant exposure taking into account other confounding variables.

Various common household activities can compromise IAQ such as heating, cooking, cleaning, use of solid cooking fuels, burning candles, or smoking, to variable extents in both developed and developing countries.^2^ Related to this, in developing countries, the energy source for heating and cooking is biomass fuel (coal and wood burning), whereas in developed countries fossil fuel (gas and liquid), in addition to electricity, are primary sources of consumable energy^2^ (Fig. 2), suggesting socioeconomic status can impact type of IAP. During heating and cooking, a wide variety of health-damaging substances can be found streaming out of a hearth fire, or stove.^2^ These include fine PM (PM2.5) or small particles (PM10), O_3_, nitrogen dioxide (NO_2_), carbon monoxide (CO), and sulphur dioxide (SO_2_)^2^*.* More specifically, UFP can be generated by biomass burning in common household activities such as heating, cooking, in addition to using printers. It is noteworthy UFP can penetrate deep into the lungs, enter the bloodstream, and travel to distant organs,^2^ thus leading to diseases like ischemic heart disease and diabetes.^58^ Interestingly, candle and incense burning, a universal social and cultural practice, emits smoke rich in CO, CO_2_, SO_2_, in addition to aldehydes precursors, among others.^59^ Also, some building specific elements can compromise IAQ by generating VOCs, an indoor pollutant present in common household items such as furniture and sanitation material but also generated by microbial growth.^60^ Formaldehyde, a secondary pollutant implicated in sensory irritation, can originate from the interaction among its precursors such as O_3_ or NO_x_ and terpenes, for example, an ingredient commonly present in hygienic products.^61^^,^^62^

## Indoor aeroallergens

Allergens when introduced into ambient air can cause sensitization, which may lead to allergic diseases and adverse health effects. These biological air pollutants are derived from living organisms, and they have well defined structural components and aerodynamic ambient distribution.^63^ Similar to outdoor and indoor chemical pollutants, some allergens are more important outdoors (grasses, trees, weeds, fungi) while others are more important indoors like mammal-/arthropod-derived allergens (cat, dog, dust mites). Molds, similar to PM, can contribute simultaneously to both indoor and outdoor illnesses.

Several epidemiological studies correlated ambient HDM and cat allergen levels with potential risk of sensitization and subsequent allergic diseases.^64^^,^^65^ Accordingly, many indoor avoidance strategies have been designed with the aim of lowering permissible levels of these allergens to prevent sensitization and allergic symptoms. Furthermore, there is ample literature on the impact of indoor allergenic pollutants on atopic diseases. From this perspective, the role of only indoor allergens as biological air pollutants will be discussed.

## The interface between chemical/biological air pollutants and respiratory disease/ocular diseases

**Fig. 3:**
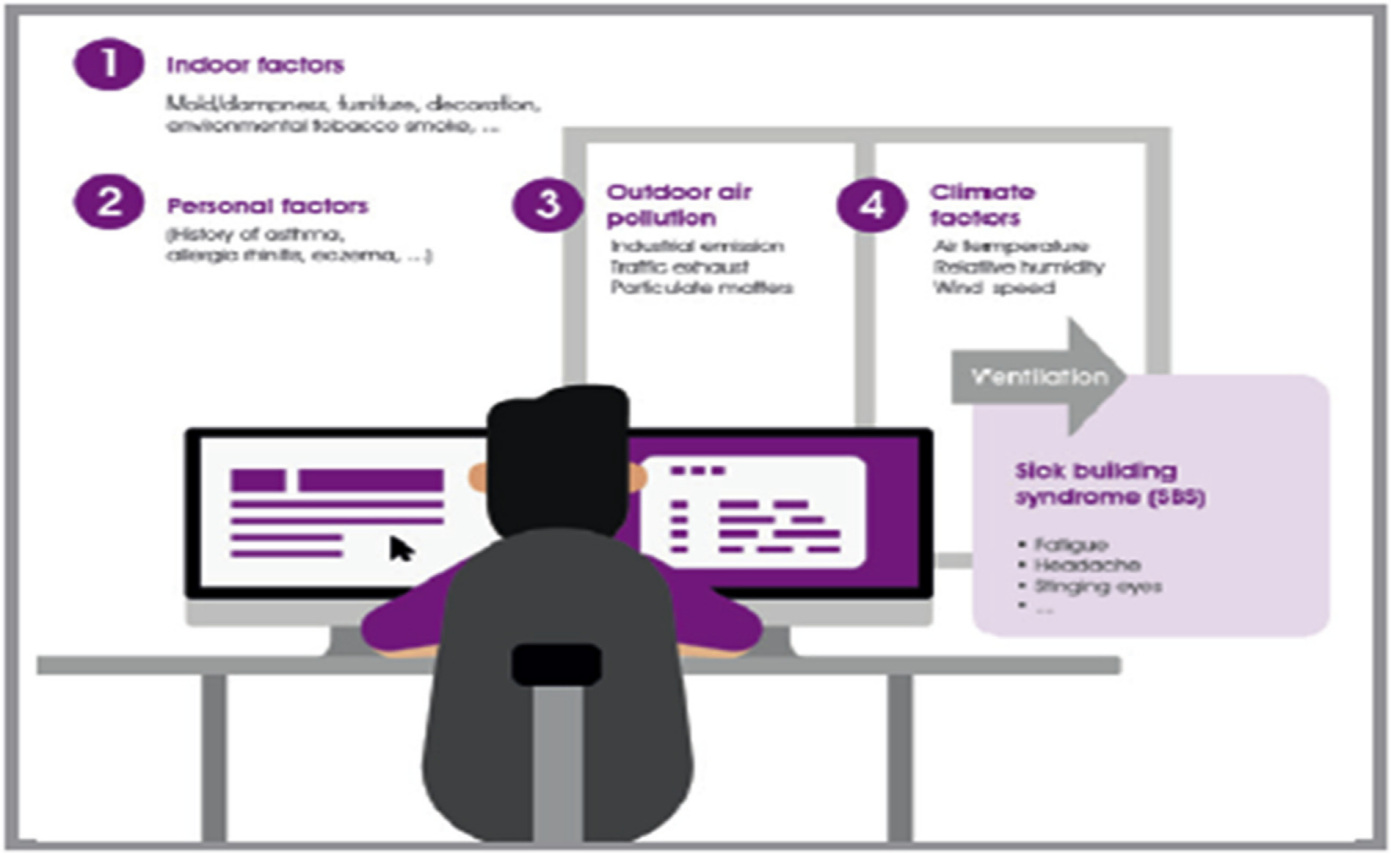
Factors involved in phenotypic expression of AR, NAR, and asthma. Adapted with permission from Lu C, Deng Q, Li Y et al. Sci Total Environ. 2016, 1:560–561:186–196

As mentioned earlier, the impact of pollution on different phenotypes of respiratory allergic and non-allergic diseases such as AR, nonallergic rhinitis (NAR), and asthma can be modulated by: 1) various indoor activities (e.g. furniture and decorations, mold and dampness, and environmental tobacco smoke); 2) outdoor proximity to heavy emissions sources like highways and industrial zones; 3) pre-existing respiratory condition; and 4) various climate factors (e.g. ventilation and wind speed, air temperature and relative humidity) (see Fig. 3).^66^ When these various factors coexist, the clinical scenario becomes a challenging one, such as occurs in an AR patient manifesting signs of worsening rhinitis in the presence of known irritants and pollutants such as passive smoking, traffic-related emissions, or building related illness (fatigue, headache and upper respiratory tract irritation), among others. Lacking a full mechanistic understanding of how environmental pollutants and irritants can impact AR symptoms, a recommendation to minimize or avoid individual exposure, if possible, might prove beneficial to the patient.

### Epidemiological *pollutant* exposure and impact on health

An in-depth analysis of the impact of pollutant exposure on health is inconclusive based on data from experimental or short-term and long-term epidemiological exposure. Experimental and acute short-term exposure, especially close proximity studies to sources of traffic pollution, suggest an increased risk of respiratory symptoms exacerbations, while community-level long term exposure (PM10) studies failed to demonstrate a positive association with prevalence of childhood asthma and rhinoconjunctivitis symptoms.^67^

In the upper airways, numerous studies in multiple settings have shown the impact of pollution on rhinitis. In a large epidemiological study from Brazil, children living in polluted areas, when compared to those living in non-polluted areas, reported a higher incidence of rhinitis (7% versus 4%).^26^ Sanitation workers exposed to pollutants demonstrated significant increased inflammation and respiratory symptoms compared to controls.^68^ Following the reunification of Germany, declines in air pollutant levels in Eastern Germany were correlated with a decrease in questionnaire-based respiratory tract symptoms.^69^ In general, environmental and occupational pollutants irritate the nasal mucosa leading to the release of inflammatory mediators and augmented nasal hyperreactivity which overlaps with symptomatology of atopic diseases like AR.^70^ Pollution can involve the ocular system, although inconsistently and non-specifically. Symptoms vary from tearing, redness, to blurring of vision. However, the eyes can sometimes overcome the ongoing inflammatory surface changes through its own homeostatic defenses and thus become asymptomatic. Inflammatory mechanisms of ocular impairment can include direct surface toxicity and oxidative stress.^71^

### Epidemiological exposure studies of *aeroallergens* and their impact on health

#### Molds

Molds are ubiquitous dwellers of both indoor and outdoor environments and exhibit a distinct climate and habitat (indoor/outdoor environment) preference.^72^ Fungal colonies can release antigens or toxins in the form of fungal fragments, 0.03 to 0.3 μm in size. Indoor water-damaged environments can represent a health hazard because of the release of mycotoxins (aflatoxins, ochratoxin A, and macrocyclic trichothecenes), in addition to VOCs. This can result in specific respiratory tract diseases, central and peripheral neurological deficits, or non-specific illnesses like chronic fatigue syndrome (CFS).^73^ Targonski et al. reported that a concentration of *Alternaria* molds higher than 1000 spores per cubic meter increased asthma deaths in Chicago two-fold.^74^ Outdoor molds, *Penicillium* and *Alternaria,* and indoor dust borne *Cladosporium,* are risk factors for the development of AR. Molds may also be responsible for NAR, where there is a local reaction to mycotoxins and other irritating substances.^75^ Longitudinal epidemiological data between 2006 and 2017 suggested a cause-and-effect relationship as well as an association between exposure to molds and development and exacerbation of asthma in children, and AR, respectively.^76^ Using objective measures such as aeroallergen wheal area for detection of early sensitization in children, and nasal eosinophils as a marker of allergic eosinophilic rhinitis (AER), *Penicillium* wheal area (and sensitization) strongly correlated with AER in children at age 4, but not at age one or two.^77^ Also, in a birth cohort study, using a mold-specific quantitative PCR (MSQPCR) analysis for detection and measurement of common indoor molds, exposure to *Aspergillus* and *Penicillium* during infancy correlated with childhood asthma at age 7.^78^

#### House dust mites (HDM)

Both climate type^79^ and urbanization can alter HDM-associated atopic diseases (see Fig. 2). The cardinal source of HDM derives from household dust, but it can be detected also in bed linen, floor carpeting, furniture, and infrequently washed clothing.^80^ Overall, the majority of residencies in the United States (up to 84%) harbor HDM at variable concentrations, 50% of which manifested concentrations beyond sensitization level, namely at 2 μg/g of dust.

During the first 3 years of life,^65^ exposure to relatively high levels of HDM allergen measured in carpet samples predisposed to sensitization to these indoor allergens. Moreover, early sensitization to HDM in newborns^80^^,^^81^ and school children^82^ is associated with asthma later in life. These data and other epidemiological studies incur an early management strategy for infants and toddlers to prevent asthma and AR.

## Impact of climate change on respiratory allergies

Anthropogenic emissions of the greenhouses gases gradually expanded over the past decades and thus increased global temperature. This major change has an enormous effect on the planet including the human environment.^21^ The impact of climate change on respiratory allergies is a complex process which utilizes not only meteorological variables but also their effect on airborne allergen behavior and evolving ambient pollution.^83^ The allergenic load of pollen and spores is produced and dispersed in an altered way commensurate with climate change and with more deleterious outcome on allergic patients. In addition, climate change has immediate and long-term effects on inflammation and infections of the airways. The increased atmospheric pollen concentration can be due to several factors: an earlier start of greenness pollination process leading to longer pollen seasons, a more rapid growth of some plants, increased flowering, and an increase in pollen allergen potency.^21^ These changes cause longer periods of suffering for patients with AR and asthma as well as deterioration of symptoms and consequently higher consumption of medications due to length and severity of symptoms. Exposure to cold dry air with air conditioning systems mimics climate change on a microscale and results in significant public health issues.^84^

Thunderstorms as well as heavy rainfalls are particularly important climate conditions because they can result in an increase in atmospheric concentration of pollen-incorporated allergenic particles of respiratory size, hypothetically secondary to osmotic shock-induced pollen rupture,^85^ thus releasing allergen-carrying paucimicronic particles. The temporal association of thunderstorms with epidemics of asthma outbreaks, sometimes near fatal, has been highlighted epidemiologically in pollinosis patients during pollen season whilst, under the same conditions, most thunderstorms are not followed by large asthma epidemics. Although atmospheric pollen level correlates with severity of asthma symptoms, the relationship between exposure to high pollen count, the nature of airway inflammation, and clinical symptoms needs further investigation.^83^

Attempts to mitigate the repercussion of climate change on our planet will require measures to reduce global emissions. Until then, humans will need to adapt to the impact of future climate variability.

## Mechanisms by which air pollution aggravates allergic rhinitis

### Mechanism of IgE mediated hypersensitivity

The pathophysiology of AR is well elaborated. Exposure to allergens in atopic individuals leads to the development of specific IgE antibodies which come to reside on mast cell surfaces as well as other cells. In a sensitized individual, re-exposure to allergens activates mucosal mast cells to release histamine, arachidonic acid metabolites (sulfidopeptide leukotrienes, prostaglandins), and other vasoactive and inflammatory mediators, resulting in itching, sneezing, nasal secretion, and vascular congestion. Following the acute response, an inflammatory response occurs creating late-phase symptoms manifested over hours. Repeated exposure, as occurs during seasonal exposure, leads to increasing tissue inflammation which may last for days. The inflammation changes the reactivity of the nasal mucosa to further exposure to allergen and nonspecific irritants.^86^

### Mechanism of oxidative stress in healthy humans and animals

Animal and human studies have looked at objective and subjective nasal responses to environmental pollutants in controlled exposure protocols. Using a sino-nasal exposure model in healthy mice, oxidative stress pollutant exposure induced a Th2 like inflammatory profile in nasal lavage fluid.^87^ In the upper airways, controlled exposure of healthy individuals to VOCs showed increased nasal symptoms score for irritation and odor intensity^88^ and an increase in polymorphonuclear leukocytes (PMNs) in nasal lavage.^89^ In the lower airways, an early neutrophilic, macrophages, and monocytic inflammation followed exposure of healthy volunteers to ozone.^90^ These data highlight the ability of ambient particulates to elicit an antigen-independent allergic inflammation.

### Mechanism of oxidative stress in allergic vs healthy individuals

Allergic individuals can have different responses to pollutants compared to a healthy population. In fact, patients with seasonal AR exposed to chlorine gas in a climate-controlled chamber had more nasal congestion compared to non-rhinitis patients.^91^ In the lower airways, studies show that ozone challenge shows greater increases in neutrophil and eosinophil levels in allergic asthmatic individuals compared to non-allergic asthmatics.^92^ This has also been seen by gene expression studies when cells recovered from sputum of healthy volunteers and allergic asthmatic patients were challenged with ozone.^93^ The gene expression profiles of cells from these 2 populations were shown to be notably different, particularly for genes involved in the ERBB2 pathway (ERBB2, MMP2 and CCND1), the antioxidant response (NQO1, GSTM4, GPX3 and NCF2), and innate immunity (HLA-DPA1, ICAM1, IL-6, IL-8, IL-18 and TNFα).^93^

### Mechanism of oxidative stress in AR patients

Data suggest environmental pollutants can act synergistically with allergens to enhance allergic response in atopic individuals. For example, when preceded by allergen challenge in the lower airways of atopic individuals, the allergic inflammation resulting from inhalation of DEP was amplified. This was manifested by increased recruitment of inflammatory cells (neutrophils and eosinophils), amplification of cytokine production (IL-5 and IL-8), and inflammatory biomarkers such as eosinophil cationic protein (ECP) and monocyte chemotactic protein (MCP)-1.^94^ In the upper airways, controlled exposure to ozone or VOCs to healthy individuals results in recruitment of neutrophils into the tissues, suggestive of Th1 profile; whereas in the lungs ozone exposure to healthy or allergic individuals results in both neutrophilic and eosinophilic recruitment, suggesting a mixed Th1, Th2 profile. The majority of studies on DEP exposure seems to skew the immune response towards a Th2 profile.^95^, ^96^, ^97^

The aforementioned experimental studies suggest the nature of recruited inflammatory cells and expression pattern of different cytokines following pollutant exposure vary according to the studied population (animal model, healthy volunteers, or allergic patients), respiratory organ under study (lung or sino/nasal tissues), type of exposed pollutant (PM, ozone, or DEP), and experimental protocol (involving or not an allergen challenge prior to pollutant exposure). Improvising novel and standardized human and animal experimental models to study pollutant exposure will shed more light on the nature of the involved immunological profile.

### Neurogenic-mediated mechanism

Environmental pollution can trigger rhinitis via a neurogenic mechanism. It is mediated by the trigeminal nerve whose peripheral ending chemoreceptors have inherent ability to detect both aerosol irritants and bitter tastants.^98^ This neurogenic mechanism of environmental rhinitis (a non-allergic rhinitis phenotype) is suggestively mediated through a local axon reflex, a central autonomic reflex, mast cell degranulation, or epithelial cell activation.^99^ The resultant upper and lower airway response to irritant exposure manifests as rhinorrhea, nasal obstruction, sneezing, coughing, and laryngospasm. The responses to air pollutants and allergens are not mutually exclusive, and, in fact, can influence one another.^100^ Air pollutants, in addition to their neurogenic irritant property, can cause unpleasant smells by triggering olfactory chemoreception. Prolonged exposure to a common trigger, however, modulates olfactory and trigeminal chemoreception differently by fading out (i.e., adapt) in the former and building up with the latter, respectively.

### Mechanism of oxidative stress mediated by immunomodulation

Recent data suggest air pollutant exposure has an immunomodulatory role not only on innate immunity but also on adaptive immune response as well, and this can be mediated by important pro-inflammatory cytokines such as IL-33 which belongs to IL-1 cytokine superfamily.^101^^,^^102^ By virtue of its ability to induce oxidative stress in airways mucosal cells, PM caused enhanced expression of IL-33 in lung tissues in an animal model, in parallel with an increased allergic inflammation of the airways, partly expressed as recruitment of eosinophils and neutrophils in airway tissues and bronchoalveolar lavage.^103^ IL-33 can drive production of Th2-associated cytokines such as IL-4, IL-5 and IL-13, all relevant to allergic inflammation.^104^^,^^105^ These Th2 cytokines and their response can thus be generated by a non-allergic stimulus. This may serve as a principal mechanism of cross-interaction between the innate and adaptive immune response. Interestingly, such a cross-interaction is featured in the pathogenesis of airway inflammation, such as asthma^106^^,^^107^ and CRS.^108^

In the same context, both adaptive and innate immunity can be modulated by PM with consequent adverse outcomes on respiratory infections. In fact, epidemiological data suggest an increased or decreased incidence of respiratory infections when level of ambient pollutant is increased or decreased, respectively.^109^ One plausible mechanism is alteration of toll like receptor (TLR) signaling directly by an unknown component of pollutants, or indirectly by elevated intracellular proteins normally released by damaged cells, namely the damage-associated molecular patterns (DAMP).^110^ Distinct pollutants can trigger TLR signaling differently. For example, PM and ozone alter TLR signaling with the intermediary of DAMPs and bacterial cell wall components (endotoxin), respectively. This may result in interference with production of Th1- or Th2- related cytokines; suppression of a proper immune response against viruses, or alternatively compromising the ability of TLR to respond to DAMPs, or pathogen-associated molecular patterns (PAMPs), by pollutants acting themselves as ligands. As such, this may result in increased susceptibility and severity of viral infections. Overall, exposure to pollutants may prime and modulate the immune system response to further stimuli and thus may contribute to the pathogenesis of other respiratory illnesses such as asthma.

Taken all together, the immuno-modulatory changes which air pollutants exert on respiratory diseases include recruitment of neutrophils and eosinophils at mucosal airway barrier, nonspecific airway reactivity, increased IL-33 expression, secretion of DAMP that activate and boost the response of the innate immune system, increased IL-1β and decreased IL-10 production, and enhanced response to inhaled allergens and primary allergy sensitization.^93^^,^^103^^,^^111^

### Mechanism of pollutant-induced allergic hypersensitivity

Animal and human experimental protocols involving exposure and allergen challenge can shed light on the mechanism of pollutant-induced enhancement of upper and lower hypersensitivity in allergic patients. In one study involving atopic rhinitis and asthmatic patients, controlled ozone exposure resulted in significant deterioration of lung function (FEV_1_) when measured during early and late-phase responses to allergen challenge, mediated by an increase in sputum lactate dehydrogenase (LDH), neutrophils as well as eosinophils.^112^ Similarly, following ozone exposure, allergic subjects expressed enhancement of the late-phase response to nasal allergen challenge.^42^ In controlled animal studies, ovalbumin (OVA) sensitized mice show significant increase in bronchoalveolar lavage (BAL) eosinophils upon intraperitoneal OVA challenge and ozone exposure.^113^ Recently, in a similar animal model, allergen and ozone challenge resulted in deterioration of lung function (as measured by airway hyperresponsiveness and lung compliance), exaggerated inflammation (as measured by histopathological examination, recruitment of inflammatory cells, increased TNF-α and IL-5 level), as well as increased expression of oxidative stress markers in lung homogenates (as measured by malondialdehyde content and the glutathione peroxidase anti-oxidant activity).^114^

In summary, pollutants are not only responsible for a variety of other health-related outcomes many of which are linked to inflammation, but also exacerbate allergic airway diseases. This should trigger the clinician to investigate for environmental and occupational hazards which can impinge on the manifestations of common upper and lower airway disorders such as eustachian tube dysfunction, laryngeal dysfunction, and asthma.

## Oxidative stress hypothesis

**Fig. 4:**
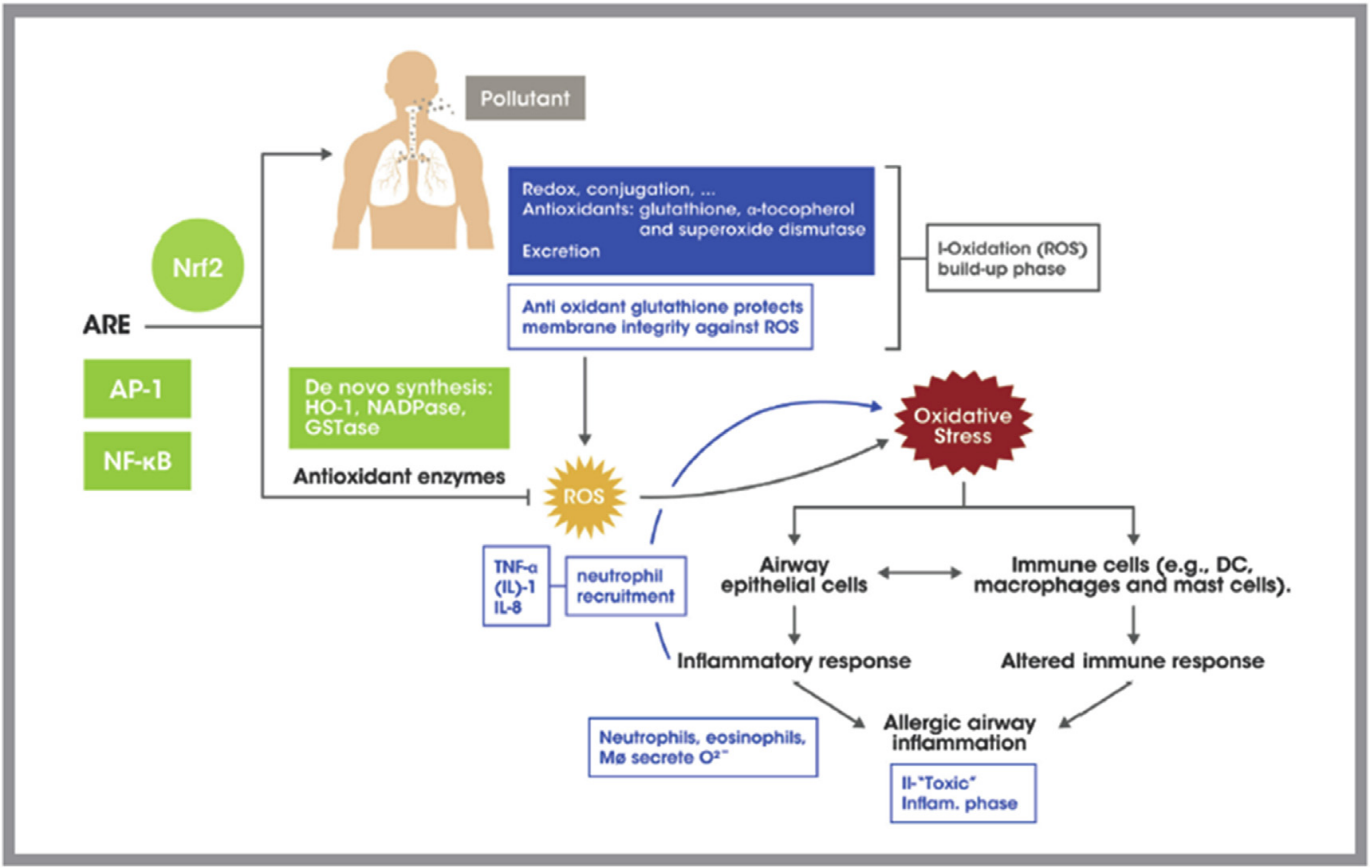
Generation of oxidative stress by pollutant: role in allergic airway inflammation, Adapted from Li N. et al. J Allergy Clin Immunol. 2016, 138, 2: 366–396.^35^^,^ ARE: Antioxidant response element; Nrf2: Nuclear Factor (erythroid derived 2) like 2, AP-1: activator protein-1, NF-κB: Nuclear Factor–kappa beta, HO: Heme oxygenase, GST-ase: glutathione S transferase

The oxidative stress hypothesis speculates that a pollutant-generated oxidant overload will result in accumulation of reactive oxygen species (ROS) and reactive nitrosative species (RNS) which ultimately leads to tissue inflammation and cell apoptosis (see Fig. 4). The proposed non-IgE mediated allergic airway inflammation occurs following exposure to pollutants and can be divided into a *non-inflammatory* and an *inflammatory* oxidative phase. The *non-inflammatory* phase is triggered by the pollutant itself and the redox reactions needed for its metabolism and elimination. These reactions result in ROS production and the activation of the constitutive antioxidant machinery (glutathione, superoxide dismutase). If the ROS load is too high, the existing antioxidant mechanisms are not sufficient to neutralize it. Then *de novo* synthesis of detoxifying enzymes, such as hemeoxygenase (HO-1) and glutathione S-transferase occurs.^115^ This activated gene expression is mediated by antioxidant response element (ARE). The transcription factors involved in oxidative stress response are - Nrf2 (Nuclear factor erythroid 2-related factor 2), AP-1 (activator protein 1), and NF-κβ (Nuclear factor-kappa β).^116^^,^^117^ Furthermore, if anti-oxidation mechanisms are not sufficient to neutralize ROS, then the *inflammatory* phase of the oxidative stress takes place. Now immune cells (macrophages, neutrophils, and eosinophils) are recruited through the effect of pro-inflammatory cytokines (TNF-α) and also become activated to produce ROS, therefore contributing to the oxidative stress propagation.^118^^,^^119^ Direct consequences of increased ROS production include the peroxidation of lipids and oxidation of proteins which lead to aberrant activation of signaling pathways, compromised integrity of plasma and nuclear membranes, and DNA methylation (epigenetic changes).^120^ All these events precede the allergic inflammatory response in the airway which closely resembles the Th2 IgE-mediated hypersensitivity response. Air pollution enhances response to inhaled allergens and primary allergy sensitization. In that sense, air pollution can potentially act as an adjuvant for AR.^121^

Oxidative stress has been well described in the pathogenesis of AR, asthma, and chronic obstructive pulmonary disease (COPD). Theoretically oxidative stress-induced antioxidant enzymes, such as superoxide dismutase (SOD), Glutathione peroxidase (GSH-Px), and paraoxonase (PON-1), among others, can be used as biomarkers of oxidative damage in healthy and allergic patients. In one study, plasma PON-1 (as measured by paraxon hydrolysis) was significantly decreased in HDM-AR patients compared to controls. Simultaneously, plasma total oxidative status (TOS) was increased and both PON-1 and TOS correlated with clinical severity of AR (as assessed by nasal symptoms score) independently of serum total or specific IgE level. This suggests both PON-1 and TOS can be used as allergic as well as non-allergic inflammatory markers.^122^ In another study, erythrocyte levels of SOD (as estimated by its metal components, copper and zinc) but not GSH-Px, was significantly decreased in AR allergic patients as well as atopic and non-atopic asthmatics when compared to controls. In contrast, erythrocytes levels of malondialdehyde (indicative of lipid peroxidation) remained unchanged, suggesting oxidative compensation by different constitutive antioxidant machinery.^123^

## Management of Allergic Rhinitis aggravated by air pollution

### Clinical history and physical examination

Clinical history is of prime importance in diagnosing AR, determination of its severity, and patient's feedback on previous ancillary anti-allergic therapy such as antihistamines and intranasal steroids (INS). Patients with AR suffer from sneezing, watery rhinorrhea, itching of the nose, throat, and palate, and nasal obstruction, in addition to ocular symptoms. Congestion is commonly the most bothersome symptom in AR patients but can be observed in many other sino-nasal disorders including septal deviation, adenoid hypertrophy in children, acute and chronic rhinosinusitis (RS), side effects of topical and systemic medications, and septal perforations. There are no specific symptoms that differentiate AR aggravated by air pollution from other sino-nasal disorders.

### Clinical tests to collaborate in allergy

IgE mediated hypersensitivity can be tested by serum sampling or skin testing. Both methods represent reliable, sensitive, and specific means of allergen-specific IgE determination and confirmation of atopic state. Nevertheless, interpreting complex results of both testing methods requires a skilled specialist.^124^ There are no tests to identify air pollutants.

### Non-pharmacological

#### Avoidance

Air pollution monitoring is essential. Along this, the Clean Air Act (USA-1970) aimed at improving air quality standards by regulating emissions of hazardous air pollutants. The acceptable levels of several air pollutants including CO, NO_2_ and SO_2_, were steadily decreased overtime.^1^ Education to increase awareness of the impact of pollution to all stakeholders, involving public alertness, reducing exposure to pollutants and increasing compliance to recommendations to reduce pollutants, are all crucial. Abatement of the risk factors for respiratory disease, in particular, tobacco smoke, indoor biomass fuels, outdoor pollutants, urban planning (house–interstate distances), and national and international regulation will achieve health benefits.

Allergen avoidance, where possible, is recommended, though little data support this common sense recommendation. Use of nasal filters and nasal blockers (as nasal mucosal barrier) could be a useful alternative for some individuals.

Central heating, ventilation and air conditioning (HVAC) systems have the inherent capacity to circulate an extensive air volume within residences and, consequently, can reduce indoor air pollutants by improving outdoor and indoor air exchange. These can be combined with high efficiency particulate air (HEPA) filters capable of extracting 0.3 μm in size air particles and can effectively reduce infectious and allergic triggers of respiratory illnesses.^125^^,^^126^ An alternative or better, a supplement to this, is opening windows periodically to allow external air ventilation, if outside temperature, humidity, and level of outdoor pollutants permit.^125^^,^^126^ Nevertheless, current evidence strongly suggests air filtration is a powerful tool to reduce the health risks of PM exposure and prevent disease progression.^127^ Other filters called powered electronic filters use high voltage electrical field to ionize particulates which then can get deposited downstream on collecting panels within the device. Unfortunately, they generate ozone. A list of Suggestions to Create a Healthy Environment is provided in a section at the end of the article.

### Pharmacological

If both AR and pollutants cause end-organ hypersensitivity, patients can manifest the combination of both. Thus, reducing AR induced hypersensitivity will reduce the overall hypersensitivity symptoms of the patient with allergic rhinitis aggravated by air pollution.

Management of AR alone is well elaborated and should follow guidelines,^124^^,^^128^, ^129^, ^130^ taking into consideration the recently suggested principles of precision medicine.^131^ Despite recent discoveries on mechanistic biomarkers and signal pathways of cellular oxidative stress injury secondary to pollutant exposure, efficacy studies on pharmacological therapy of AR patients exposed to specific pollutants is currently lacking. However, fexofenadine, a non-sedating second-generation antihistamine, demonstrated efficacy and a well-tolerated safety profile in ragweed AR patients exposed to ragweed associated to DEP in an environmental exposure unit. In this placebo-controlled study patients underwent ragweed allergen challenge outside their allergy season and had improved nasal symptom scores following ragweed plus DEP exposure when pre-treated with fexofenadine compared to the placebo group.^132^^,^^133^ Further studies are needed to assess efficacy of other conventional AR pharmacotherapy (e.g., intranasal steroids (INS)) in lowering oxidant overload in patients suffering from both AR and exposure to various air pollutants.

#### Antioxidants

Oxidative stress following exposure to pollution triggers endogenous antioxidant mechanisms in respiratory epithelial cells. Epidemiological data suggest intake of exogenous (dietary) anti-oxidant may decrease prevalence of atopic disease.^134^ Exogenous anti-oxidants have been studied extensively *in viv*o and *in vitro* animal models,^135^ but their efficacy in improving health and preventing illness remains to be demonstrated clearly. Despite this, individuals with marked deficiency or poor access to dietary antioxidants and highly exposed to environmental sources of oxidants might benefit from dietary antioxidants in the form of vitamin supplements.^136^

### Recommendations for patients

Unequivocally, sources of air pollution should be addressed formally with policies to reduce their emissions. However, individual actions can mitigate effects of pollution on health. The following recommendations can be emphasized to the general population, more so to individuals especially vulnerable to the detrimental effect of pollution, namely elderly people, patients with significant cardiopulmonary diseases, and children.^21^^,^^137^^,^^138^

• Remain indoors with windows closed during high pollen level or during the start of a thunderstorm
• Avoid tobacco in all forms of inhalation
• Avoid burning incense and candles
• Avoid domestic spray and other cleaners
• Eliminate source of indoor mold spores (water-damaged ceilings, walls, carpet, and furniture, especially after flooding) or thoroughly cleaning with solution containing hypochlorite
• Substitute contact lenses for daily-disposable lenses in patients with conjunctivitis.
• Use second generation non-sedating antihistamines or intranasal corticosteroids (INS)
• Use anticholinergics when clear watery rhinorrhea is a problem
• Rinse with nasal lavage solutions to conceptually minimize pollutant exposure
• Adjust treatment according to meteorological forecasts and indoor/outdoor pollutant levels including allergen levels (i.e. pollen and fungal spores)
• Avoid interventions with unintentional disadvantageous health consequences. For example, to avoid exposure to aeroallergens and air pollutants, one may reduce outdoor physical activity and exercise, thereby contributing more to the risk factors of cardiovascular and pulmonary diseases.^139^

To help in building a healthy environment:

1. Information of early signs of air pollution at individual and at population level should be provided, in particular to vulnerable and at-risk populations.
2. Sensors to measure the level of air pollutants and alert the allergic respiratory sufferers could be useful to better control symptoms.
3. Use of a daily digital record-type of mobile application for daily monitoring of allergy symptoms can help identify the relevant and manageable air pollutants.
4. Markers (ocular, nasal, and asthma) can be used as early health indicators of risk for significant morbidity following pollutant exposure in selected individuals (e.g. asthmatic children and elderly persons with respiratory illnesses).

## Precision medicine

In precision medicine management is focused on a predictive, preventive, participatory, and personalized (the 4 Ps) basis, therefore, a holistic approach to the evaluation of AR should encompass the following principles: 1) Risk assessment for developing allergic diseases and their exacerbation; 2) Comprehensive evaluation of the environment-related risk factors contributing to the disease burden in that patient; 3) Reduction of individual-based and environment-related risk factors by appropriate interventions and periodic checks of their effectiveness; 4) Promotion of preventive interventions among patients through effective communication (e.g., social media) in order to improve compliance to these interventions.^140^

Proper implementation of the concepts of precision medicine in the management of pollution health related effects in allergic patients is currently lacking. Attempts to improve our understanding should encompass the following general approaches: 1) Insight into gene-environment interactions (i.e. internal and external exposomes); 2) Better characterization of clinically important symptoms in order to treat or prevent them, should we be unable to prevent the disease itself; and 3) Assess clinical effectiveness of these measures, their overall cost to the individual, the health care system, and society.^140^

## Conclusion

Current evidence reveals patients with AR exposed to pollution and climate change have significant adverse health effects. Epidemiological and clinical studies demonstrate the immunological effect resulting from both aeroallergen and pollutant co-exposure, which is inducing inflammatory responses with recruitment of inflammatory cells, cytokines, and interleukins. Besides the immuno-pathogenic mechanism, rhinitis symptoms can be mediated by a neurogenic component upon exposure to environmental irritants. As well, human experimental studies involving specific pollutant exposure and allergen challenge suggest pollution can exacerbate allergic airway disease and increase organ responsiveness. Despite advances in understanding mechanisms of airway inflammation, current evidence is less clear about the benefits of management of coexisting AR and pollution, especially when it comes to pharmacological therapy. Certainly, the efficacy of fexofenadine in improving allergic rhinitis symptoms in patients exposed to air pollution should lure more clinical studies on the role of other related drugs such as INS in mitigating symptoms resulting from co-exposure to air pollution and allergies. Notwithstanding, individual and carefully chosen avoidance measures, in addition to conventional AR pharmacotherapy, can alleviate rhinitis symptoms triggered by AR and air pollution.

## Unmet needs in the management of allergic rhinitis in the setting of air pollution

We currently lack understanding of how the collective environmental exposure, whether specific or non-specific, can influence cellular responses in allergic patients across an individual's life cycle. In particular, we must understand the relationship among weather variables, air pollutants, aeroallergens, and the mucosal barrier. This intricate association is influenced further by genetic polymorphism and epigenetic changes inflicted by pollutant exposure.

The "greenhouse effect" on production and distribution of biological allergens is somewhat understood, but data on the effects of air pollution on allergen content are lacking. Further studies on biomarkers that predict an effect of air pollution in AR patients are needed. Better understanding of the oxidative stress mechanism can form the basis of engineering corresponding antagonists.

In this approach we must consider that oxidative stress by distinct air pollutants may induce different inflammatory profiles in the upper and lower airways in both healthy and allergic patients. Moreover, many of the suggested environment-based strategies to reduce the impact of air pollution on AR patients (e.g., air filters and environmental behavioral modification) need to be validated for clinical efficacy as well as the levels at which air pollutants cause change in the individual.

### Ethical approval

Not applicable.

## Declaration of Competing Interest

1. Robert Naclerio: Lyra, Sanofi and Actos (Advisory Board; Speaker), Optinose.

2. Ignacio J Ansotegui.: No conflict of interest to declare.

3. Jean Bousquet: Chiesi, Cipla, Hikma, Menarini, Mundipharma, Mylan, Novartis, Sanofi-Aventis, Takeda, Teva, Uriach (Advisory Board, consultant, meeting lectures fees), Kyomed (shares).

4. Giorgio Walter Canonica: BI, ALK, Stallergens (Grant/research support), (Menarini, GSK, Sanofi, Teva, Hal, AZ, Novartis (honoraria or consultation fees).

5. Gennaro D'Amato: No conflict of interest to declare.

6. Nelson Rosario: MSD, GSK, Takeda, Sanofi, Danone, Novartis, Chiesi, AstraZeneca, Aché, FDA Allergenic, Boehringer Ingelheim (speaker); Takeda, MSD, Danone, Boehringer Ingelheim (written material); Danone, MEDA, Sanofi, Boehringer Ingelheim (Advisory).

7. Ruby Pawankar: No conflict of interest to declare.

8. David Peden: No conflict of interest to declare.

9. Karl-Christian Bergmann: No conflict of interest to declare.

10. Leonard Bielory: Allergy Therapeutics, Sanofi, Chattem (consultant), US EPA, CDC (Granta).

11. Luis Caraballo: No conflict of interest to declare.

12. Lorenzo Cecchi: No conflict of interest to declare.

13. S Alfonso M Cepeda: No conflict of interest to declare.

14. Herberto Jose Chong Neto: No conflict of interest to declare.

15. Carmen Galan: No conflict of interest to declare.

16. Sandra N Gonzalez Diaz: No conflict of interest to declare.

17. Samar Idriss: No conflict of interest to declare.

18. Todor A Popov.: No conflict of interest to declare.

19. German Dario Ramon: No conflict of interest to declare.

20. Erminia Ridolo: Faes Pharma (speaker).

21. Menachem Rottem: No conflict of interest to declare.

22. Wisuwat Songnuan: No conflict of interest to declare.

23. Philip Rouadi: GSK, MSD, Astra Zeneca, Bayer, Takeda, Novartis, Meda, Pharmaline, Algorithm, Abbott, Sanofi Pharmaceuticals (Speaker's Desk).

## **Suggestions to Create a Healthy Environment**

### **Reduce allergens in your home**.

Common indoor objects and surfaces that can harbor allergens such as dust mites, molds, and pet dander, and trigger indoor allergies, may include the following:

• Toys such as stuffed animals can conceal dust mites, molds, and pet dander.
• Clothes when left on the floor or kept for a substantial time in drawers can enclose allergens.
• Carpets contain relatively high concentrations of allergens especially molds (wall-to-wall carpeting) because they can be difficult to keep dry. They can be substituted for rugs provided they are frequently washed and vacuumed.
• Hard surface flooring can substitute carpeting, but care should be taken to minimize exposure to indoor pollutants when waxed or vanished (choose cleaning product with low VOC by talking to manufacturer) in order to reduce symptoms of rhinitis.
• Furniture and pressed wood can emit high levels of VOCs and formaldehyde, respectively.

### **Ventilate your home**.

As recommended by the U.S. Environmental Protection Agency (EPA), home ventilation is important to reduce indoor air pollutants by exchanging air in outdoor and indoor dwellings. It should be performed on a daily basis, frequently during the day for up to 10 minutes each time.

• Home ventilation should concur with certain indoor activities such as cooking where it is also advisable to use an extractor hood, or during shower time to minimize moisture in the bathroom or bedroom.
• Interior decoration and wall paintings require proper home ventilation.
• Modern HVAC systems, ideally integrated with a HEPA filter, can ensure efficient home ventilation provided filters are regularly cleaned. Short of this, opening windows periodically to allow external air ventilation is recommended, if outside weather conditions and pollutants level permit.
• It is advisable not to ventilate homes at times of high levels of pollen, fungal spores, or air pollutants.

### **Suggestions to Create a Healthy Environment**

*continued*

#### **Bedroom tips**

Mattresses, pillows, and blankets frequently harbor mold and dust mites which can thrive on dander of pets, if present. Allergens from these different sources can trigger allergic symptoms. This can be counteracted by:

• Washing bedding on weekly intervals using hot water at 54°C and bleach to kill dust mites and their eggs in addition to mold spores.
• Employing commercially available mite-proof bedding.
• Renewing mattresses every 8–10 years.
• Restricting pets (and their sleeping cages) from bedroom areas.
• Vacuuming bedrooms in an attempt to decrease allergen load.
• Refraining from eating in bedrooms.

#### **Smart cleaning**

Removing indoor dust aims at reducing the allergen rather than dispersing it. The choice of cleaning products should also be made wisely. Consequently:

• Feather dusters scatter dust and allergens and thus should be avoided
• Moist cloths are preferred over ordinary ones for wiping dirt. The latter can simply disturb allergens which become airborne again and float back on other indoor surfaces.
• Vacuum cleaners should be sealed tightly to avoid dust leak, whether incorporated or not with a HEPA filter. Dust containers in vacuum cleaners should be emptied and cleaned outdoors to avoid dispersing dirt indoors.
• Cleaning products with a high composition of VOC, scents, or odors and other potential irritants can trigger an allergic reaction and thus should be avoided.
• A well experienced exterminator can advise about traps and solvents that are more appropriate to use for individuals with rhinitis or asthma.
